# Structural and Functional Differences in the Long Non-Coding RNA *Hotair* in Mouse and Human

**DOI:** 10.1371/journal.pgen.1002071

**Published:** 2011-05-26

**Authors:** Patrick Schorderet, Denis Duboule

**Affiliations:** 1 School of Life Sciences, Federal Institute of Technology (EPFL), Lausanne, Switzerland; 2 National Research Center “Frontiers in Genetics,” Geneva, Switzerland; 3 Department of Genetics and Evolution, University of Geneva, Geneva, Switzerland; Medical Research Council Human Genetics Unit, United Kingdom

## Abstract

Long non-coding RNAs regulate various biological processes such as dosage compensation, imprinting, and chromatin organization. HOTAIR, a paradigm of this new class of RNAs, is localized within the human *HOXC* gene cluster and was shown, in human cells, to regulate *HOXD* genes *in trans via* the recruitment of Polycomb Repressive Complex 2 (PRC2), followed by the trimethylation of lysine 27 of histone H3. We looked for the presence of *Hotair* in mice to assess whether this *in trans* mechanism was conserved, in particular at the developmental stages, when *Hoxd* genes must be tightly regulated. We show that the cognate mouse *Hotair* is poorly conserved in sequence; and its absence, along with the deletion of the *HoxC* cluster, has surprisingly little effect *in vivo*, neither on the expression pattern or transcription efficiency, nor on the amount of K27me3 coverage of different *Hoxd* target genes. We conclude that *Hotair* may have rapidly evolved within mammals and acquired a functional importance in humans that is not easily revealed in mice. Alternatively, redundant or compensatory mechanisms may mask its function when studied under physiological conditions.

## Author Summary

Long non-coding RNAs (lincRNA) have recently become a new paradigm for gene regulation *via* chromatin remodeling in a variety of biological processes, including during embryonic development. HOTAIR, a human lincRNA localized within the *HOXC* cluster was shown to help silence *HOXD* cluster genes in *trans*, through the recruitment of the Polycomb Repressive Complex (PRC2). In this paper, we investigated the role of the murine *mHotair* lincRNA and report that both its structure and function are quite different from that described in human cells. Deletion of *mHotair in vivo* has little effect on the transcriptional regulation and chromatin modification of mouse *Hoxd* genes, and embryonic transcriptomes did not reveal any particular effect upon genes reported as targets of PRC2 in ES cells. Our results indicate that the function of this RNA in mice is distinct from that reported for human cell lines, pointing to a rapid evolution of this lincRNA. Alternatively, redundant mechanisms may mask the function of *mHotair* in physiological conditions or this lincRNA may be required in a very restricted and specialized cell type.

## Introduction

Genomes contain a large number of RNAs, which do not encode any protein [1]–[5]. While some of these non-coding RNAs such as XIST, TSIX and AIR associate with epigenetic modifying complexes [6]–[11], the functions of others remain poorly understood. Many of the predicted long non coding RNAs (lincRNAs) are thought to be spliced and polyadenylated, thus resembling protein coding RNAs [12]–[15] and have been proposed to impact on gene regulation [16], [17].

Recent studies have shown that distinct lincRNAs are involved in diverse biological processes such as dosage compensation, imprinting or cancer metastasis [10], [18]–[20]. More specifically, they may function at the interface between DNA and its epigenetic regulation by targeting remodeling complexes to their target sites [21]. HOTAIR, one such lincRNA located within the human *HOXC* cluster, regulates *HOXD* cluster genes *in trans via* the recruitment of PRC2, a silencing complex responsible for the deposition of trimethyl groups on lysine 27 of histone H3 (H3K27me3) [10]. Knock-down of HOTAIR in human fibroblasts induced gain of expression of different members of the *HOX* family, associated with a loss of K27me3 decorating part of the *HOXD* locus in these cells [10].

In addition, HOTAIR has been shown to co-immunoprecipitate with members of the PRC2 complex such as SUZ12 and EZH2, but not with the putative PRC1 member YY1, suggesting a primary role in the initiation of silencing, rather than in its maintenance [6], [10], [21]. Subsequent studies have suggested that distinct sub-domains of HOTAIR are essential for the binding of either EZH2, or of LSD1 and that HOTAIR functions as a bridge to bring both complexes together. In the absence of these two binding domains, the epigenetic functionalities of this lincRNA are indeed completely abrogated [21].

Altogether, these results indicate that human HOTAIR is an important regulator of the *HOX* epigenetic landscape in skin fibroblasts. Given both the importance of this lincRNA in adult tissues and the critical dynamics of H3K27 trimethylation for the early control of *Hoxd* gene activation [22], we investigated its role in developing mouse embryos. Here, we describe the mouse *Hotair* cognate lincRNA and show that its complete depletion *in vivo* has no severe effect upon *Hoxd* gene activation, neither during early trunk development, nor in the course of limb morphogenesis, two sites where HOTAIR was seen expressed.

## Results

### The mouse *Hotair* lincRNA

**Figure 1:**
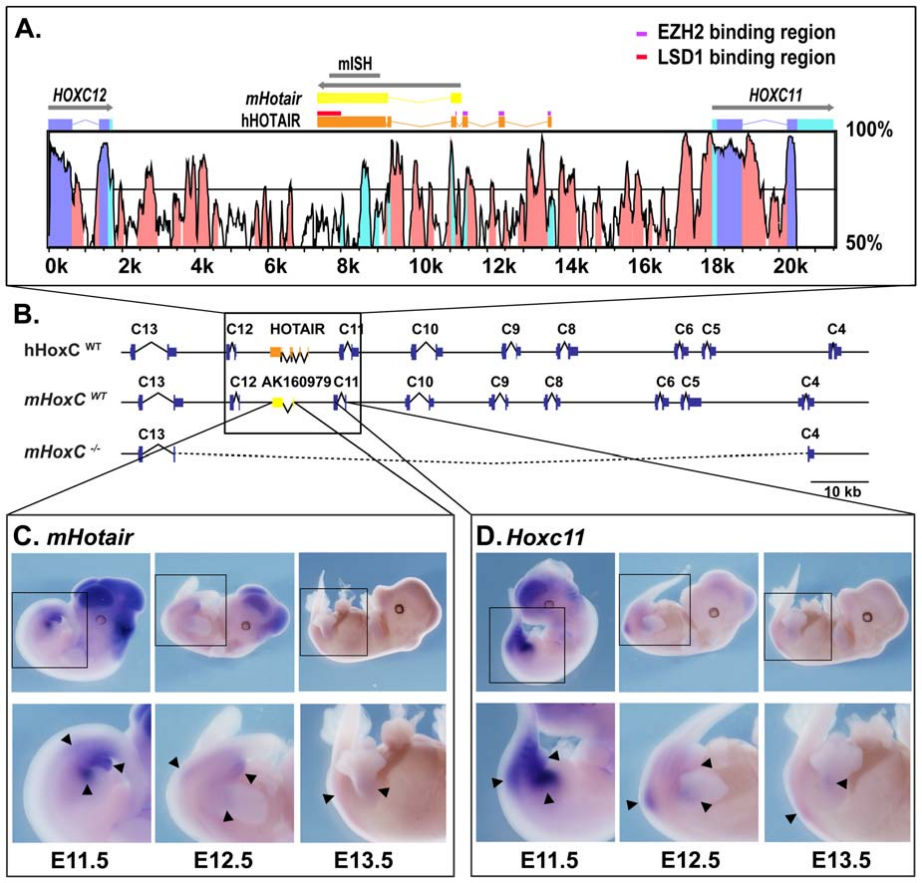
Sequence comparison and expression of *mHotair*. (A) Human-mouse sequence comparison between the highly syntenic *Hoxc12* to *Hoxc11* DNA interval, within the *HoxC* gene cluster, using the rVista software. *mHotair* is in yellow. The relative positions of HOTAIR (orange) and *mHotair* (yellow) are shown on the top. Highly conserved non-coding sequences (CNS) are shown in red and coding exons in blue. ‘mISH’ points to the genomic location of the mouse DNA fragment used as a probe for whole mount *in situ* hybridization and the previously identified sites for binding of LSD1 and Ezh2 to HOTAIR are indicated. (B) Relative positions of *mHotair* and HOTAIR within their respective gene clusters and map of the *HoxC* deficiency (bottom). (C,D) Comparative expression patterns of both *mHotair* (C) and *Hoxc11* (D), as revealed by whole mount *in situ* hybridization (WISH) on E11.5, E12.5 and E13.5 developing embryos. Panels at the bottom are enlargements of the rectangles in the panels above. *mHotair* is transcribed in the most posterior aspect of the youngest fetus, in a way similar to *Hoxc11* expression, with some more restrictions. *mHotair* transcripts are mostly detected in the genital tubercle and in the tail. The staining in the developing cerebral vesicles (in the head) is due to a frequently occurring artifactual trapping of the probe by non-opened vesicles (also present for *Hoxc11*).

We first looked for the presence of *Hotair* in the mouse genome. Because the human RNA locates between *HOXC12* and *HOXC11*, i.e. within a region of very high micro-synteny amongst all vertebrates, we performed a pair-wise sequence alignment with the cognate mouse DNA segment, using the rVISTA software [23]. Alignment of the entire mouse *Hoxc11* to *Hoxc12* region with the human genome revealed various domains of strong sequence homology (Figure 1A). Expectedly, the *Hoxc11* and *Hoxc12* exons are highly conserved, with more than 95% homology between the mouse and human sequences. However, the intergenic region between *Hoxc11* and *Hoxc12* showed more variability, with some peaks of conservation, but also segments close to random variability, as previously described [15], [24].

Sequence alignment revealed that the human HOTAIR lincRNA most likely has a mouse ortholog RNA, referred to as AC160979. This EST (*mHotair* from now onwards) is indeed located at the expected micro-syntenic position and exhibits partial homology with human HOTAIR. *mHotair* derives from the Vega Protein Coding Annotation and corresponds to the UCSC gene based on RefSeq AK035706 transcript. However, and even though *mHotair* and HOTAIR are clearly cognate transcripts, several important differences were scored. First, while the RefSeq annotation of HOTAIR indicates six exons, *mHotair* derives from two exons only. The second half of the first exon of *mHotair* seems to match exon 4 of HOTAIR, whereas the second exon clearly matches exon 6 of HOTAIR (Figure 1A). Blasts of the first three human exons against the mouse *Hoxc11* to *Hoxc12* intergenic region did not give any significant homology.

Secondly, the level of sequence similarity between different exons is highly variable. The first exon of *mHotair*, which is 234 base pairs long, shows significant conservation (>80% over >100 bp) with the human sequence. However, the second exon, which is 1770 bases long, is poorly related to the human sequence and shows conservation higher than 75% only in a sub-domain of ca. 400 bp. Altogether, this large exon, which contains the LSD1 binding region of HOTAIR, is rather poorly conserved in its mouse counterpart, ranging from 50 to 70% homology. In addition, human HOTAIR contains several binding sites for the SET domain containing PRC2 component EZH2, responsible for the histone H3 methyltransferase activity (HMTase) of this enzyme, which are absent from *mHotair*. Although it is unclear as to whether the primary nucleotide sequence or the tertiary RNA structure is involved in binding EZH2, it nevertheless suggests that the function of this RNA in mice is not identical to that described for its human cognate. Transcriptome analyses by deep sequencing confirmed that *mHotair* was most likely encoded by two exons only, instead of six in humans (see below).

### Expression of *mHotair*

*Hox* genes are clustered at four different genomic loci (*HoxA*, *B*, *C* and *D*) and are crucial in organizing the metazoan body plans. They encode transcription factors, which work in various combinations to allocate morphogenetic identities to groups of cells. To properly coordinate their transcription, these contiguous genes are activated following a collinear regulatory strategy, whereby genes positioned at the 3′ end of the cluster are activated earlier in time and more anteriorly, whereas more 5′ located genes are activated later in time and more posteriorly [25]. This sequential activation in time and space thus follows the physical positions of genes along their respective clusters. This property, which may in part depend upon chromatin modifications [22] also applies either to transgenes, when introduced into the gene clusters, or for non coding intergenic transcripts, regardless of their sense of transcription. These non-coding transcripts associated with *Hox* genes were proposed to regulate the collinear opening and maintenance of the epigenetic status of the cluster [5].

We looked at the expression of *mHotair* by whole mount *in situ* hybridization (WISH) on developing mouse embryos at embryonic day 11.5, 12.5 and 13.5, and compared with the expression of *Hoxc11*, the gene located immediately 3′ from the *mHotair* promoter. The *mHotair* probe was selected within the region showing the highest conservation with the human ortholog (Figure 1A), i.e. the middle half of the second exon, such as to compare as accurately as possible with previously published data where the distantly related human HOTAIR sequence was used as a probe for WISH on mouse embryos [10]. Experiments using sense and antisense probes confirmed that *mHotair* is solely transcribed from the opposing canonical *Hox* DNA strand, as is human HOTAIR.

As expected from its position within the ‘posterior’ part of the *HoxC* cluster, *mHotair* expression was scored in posterior and distal sites. It was readily detected in E11.5 embryos with marked staining in the posterior part of the hindlimbs, in the genital bud and in the tail. At E12.5, the expression pattern was mainly restricted to the posterior aspect of the intermediate part of the hindlimbs, as well as to the genital bud, whereas it became barely detectable at E13.5. In parallel experiments, *Hoxc11* transcripts showed a comparable distribution, yet with stronger signals at all three stages (Figure 1C and 1D), in agreement with previously published data. Given the strong similarities of expression patterns between *mHotair* and its closest 3′ neighbor *Hoxc11*, we concluded that *mHotair* is expectedly regulated in coordination with other posterior *Hoxc* genes. *mHotair* expression, however, was quite distinct from that reported in similar staged mouse embryos when using a human HOTAIR probe [10].

### Regulation of *Hoxd* genes in *trans*

**Figure 2:**
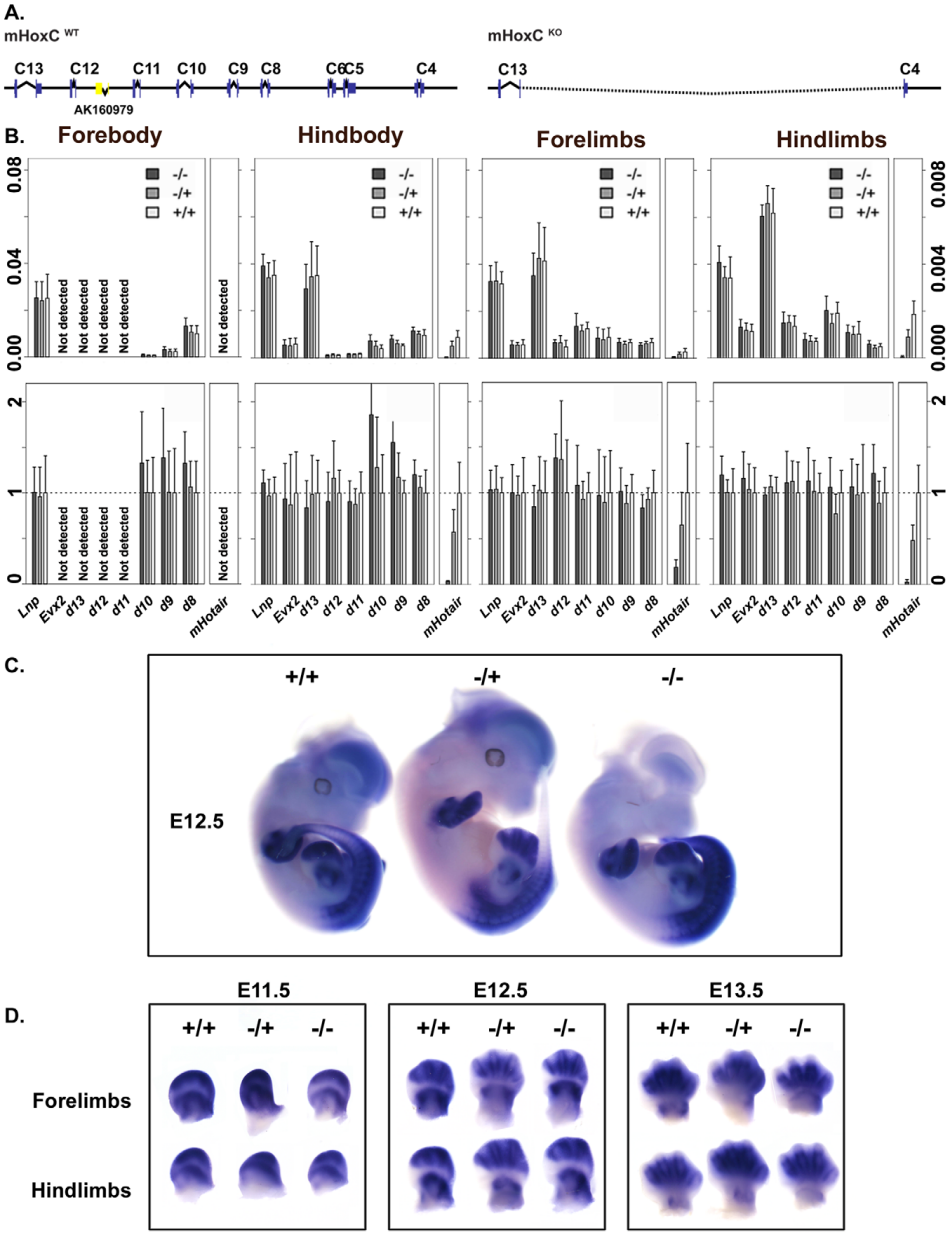
Expression analysis of different *Hoxd* genes in control and *HoxC* mutant mice. (A) Schematic representation of the wild type and the *HoxC* deleted allele. (B) Absolute and relative quantifications of posterior *Hoxd* genes transcripts and of *mHotair* in forebody, hindbody, forelimbs and hindlimbs of E13.5 embryos. All values are normalized to a housekeeping gene. Relative amounts were calculated as a ratio by forcing wild type values to 1. Accordingly, small values are over-represented, explaining why *mHotair* gives a signal after deletion of *HoxC*, even though it is obviously absent. (C) Whole mount *in situ* hybridization (WISH) of *Hoxd10* on E12.5 developing embryos. The expression domains of *Hoxd* genes remain globally unchanged (D) *Hoxd10* expression patterns in developing forelimbs and hindlimbs at three developmental stages. Expression domains of *Hoxd* genes remain globally unchanged at all stages of limb development examined.

Human HOTAIR was shown to act in *trans* by tethering Polycomb Repressive Complex 2 (PRC2) to a subset of its targets, amongst which the *HOXD* locus [10], [21]. HOTAIR thus acts as a scaffold for the repression of a number of genes in this region *via* the recruitment of these silencing proteins, with a particular impact on the expression levels of human *HOXD8*, *HOXD9*, *HOXD10*, *HOXD11* and *HOXD13*, while having no impact neither on the *HOXA*, nor on the *HOXB* and *HOXC* clusters [10]. To investigate whether this mechanism was conserved throughout mammals, we looked at the expression of these potential target genes in the absence of *mHotair*. We used a full deletion of the *HoxC* cluster whereby all *Hoxc* genes and intergenic transcripts are missing (Figure 2A) [26]. We isolated *HoxC^Del/Del^* embryos at embryonic day 13.5 (E13.5), derived from a cross between heterozygous animals, and dissected them into four distinct pieces; the forebody, hindbody, forelimbs and hindlimbs. We performed quantitative RT-PCR analyses on these various samples using wild type and heterozygous littermates as controls for homozygous mutant samples.

As expected, *mHotair* was detected neither in *HoxC^Del/Del^* mutant embryos, nor in forebody samples of all three genotypes, which we used as negative controls. In the three other samples, *mHotair* transcripts were scored, though at very low levels. However, no difference was noted in the expression levels of the presumptive *mHotair* targets *Hoxd8*, *Hoxd9*, *Hoxd10*, *Hoxd11* or *Hoxd13* (Figure 2B). The expression level of *Hoxd12* remained unchanged too, as well as those of *Evx2* and *Lunapark*, two neighboring genes largely co-regulated with *Hoxd* genes [27].

A change in the expression of different *Hoxd* genes could nevertheless remain unnoticed, should a spatial shift in their transcript patterns occur, rather than variations in their RNA steady state levels. We thus performed *in situ* hybridization on mutant animals to reveal the distribution of *Hoxd10* transcripts, which was reported as the main *HOXD* target for a HOTAIR-mediated de-repression in human cells. At all three stages examined (E11.5, E12.5, E13.5), *Hoxd10* transcripts showed wild type patterns in mutant animals (Figure 2C and 2D). Taken together, these observations indicate that *mHotair* has little or no detectable regulatory effect in *trans* over *Hoxd* cluster genes in mice, at least in these conditions.

### Tri-methylation of H3K27 at the *HoxD* locus

**Figure 3:**
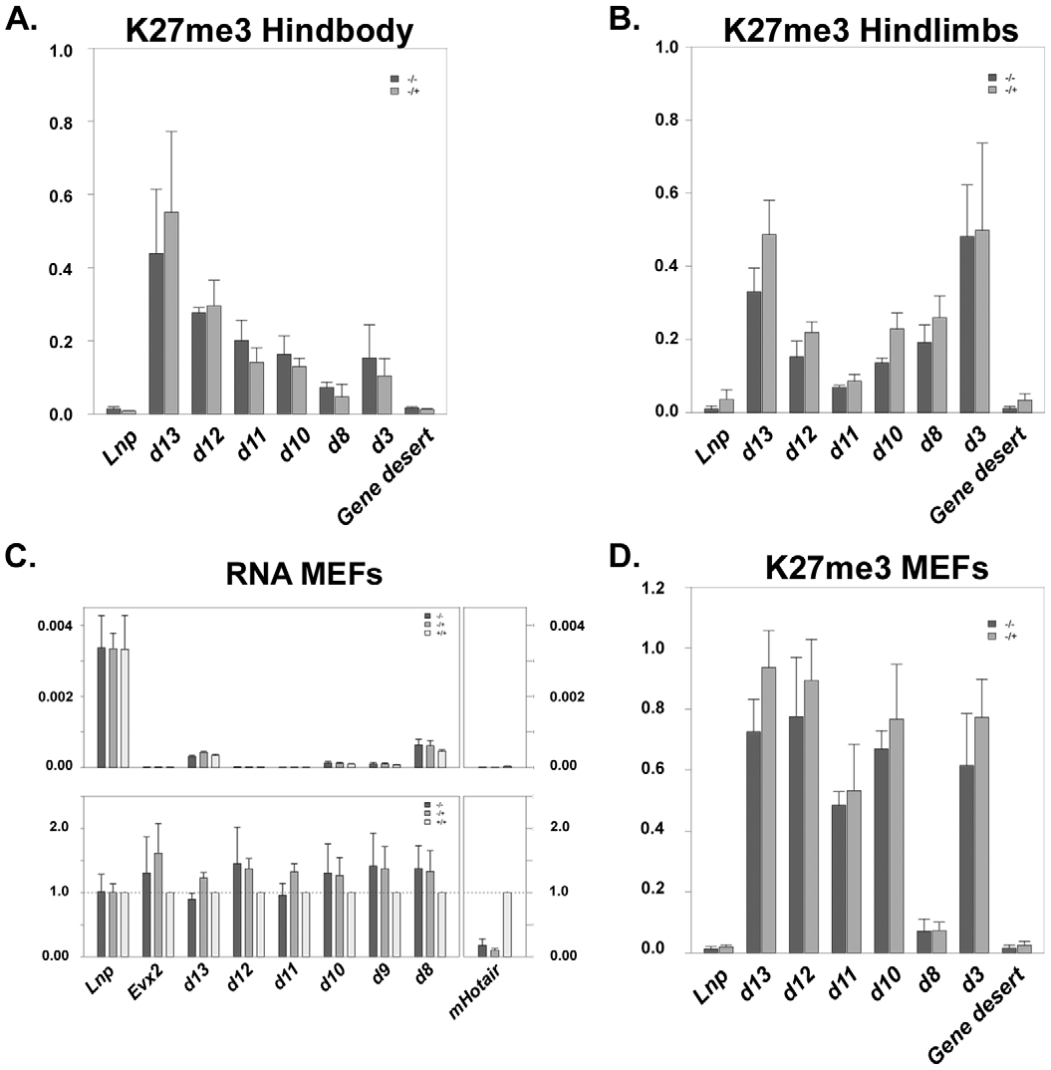
ChIP and expression profiling of control and *Hoxc^−/−^* MEFs. Enrichment of tri-methylated H3K27 over the *HoxD* gene cluster in both control mice and mice carrying a deletion of the *HoxC* cluster. The presence of this histone modification is assayed by qPCR after chromatin immunoprecipitation, either from dissected fetal hindbody (A) or from fetal hindlimbs at E13.5 (B). (C) Quantification of *Hoxd* gene transcripts present in either control, or *HoxC* mutant mouse embryonic fibroblasts (MEFs). (D) Comparison of H3K27me3 coverage between control and *HoxC* mutant-derived MEFs.

HOTAIR was reported to regulate several *HOXD* genes by tethering PcG proteins (the PRC2 complex) to the posterior *HOXD* cluster [10], [21]. Knock-down of HOTAIR in human fibroblasts indeed showed a decreased trimethylation of lysine 27 on histone H3, in particular at the *HOXD* locus, with the strongest effect observed over the region between *HOXD3* and *HOXD8*. Since a loss of H3K27me3 may not necessarily be translated into a detectable increase in *Hoxd* gene transcription in mouse embryos, we investigated the chromatin status of the *HoxD* locus in mutant animals. We used chromatin immunoprecipitation (ChIP) on E13.5 embryos, a stage at which *mHotair* is transcribed (see below), followed by quantitative RT-PCR to quantify the enrichment of H3K27me3 over the gene cluster. Here again, the parallel loss of both *HoxC* and *mHotair* alleles did not significantly alter the amount of K27me3 covering this presumptive target locus (Figure 3A and 3B). From this set of experiments, we concluded that although human HOTAIR might be essential for the recruitment of PRC2 and subsequent tri-methylation of H3K27 in cultured fibroblast, its role in the regulation of mouse *Hoxd* genes *in embryo* seems to be minor, if any, at least at this developmental stage.

### Function of mouse *Hotair* in MEFs

As the reported effects of human HOTAIR were not observed in the absence of the mouse counterpart *in vivo*, we derived mouse embryonic fibroblast (MEFs) from E13.5 embryos, either heterozygous or homozygous mutant for the *HoxC* cluster, to try and better match the conditions wherein HOTAIR's functions had been originally elucidated. We quantified both the amount of transcription of different *Hoxd* genes and the enrichment of H3K27me3 at this locus. Results obtained with MEFs heterozygous for the deletion of the *HoxC* cluster were indistinguishable from those obtained from MEFs lacking both copies of *HoxC* and *mHotair*. Analyses of both lines of MEFs gave similar amounts of *Hoxd* gene transcripts and no significant variations was scored in the enrichments of H3K27me3 marks, indicating that the presence of *mHotair* is not critical for the regulation of *Hoxd* genes in this context (Figure 3C and 3D).

### Comparative transcriptome analyses with and without *Hotair in vivo*

To assess the global impact of *mHotair* on the gene regulation, we looked at the transcriptomes of those tissues where *mHotair* was clearly transcribed at E13.5 in our whole mount *in situ* hybridization, namely the hindbody, the hindlimbs and the genital bud. Embryonic tissues were micro-dissected and total messenger RNA isolated from both control and *HoxC* mutant animals and sequenced using an Illumina Genome Analyzer. Nearly 15 million high quality single reads were mapped on the mouse mm9 genome, using Tophat [28] and visualized using the integrative genome viewer [29]. In this way, we could confirm that, as annotated in RefSeq, *mHotair* is a two-exons transcript initiating from the opposite strand of the canonical *HoxC* genes, at least in this context. No additional 5′ located exons were used, unlike in human.

**Figure 4:**
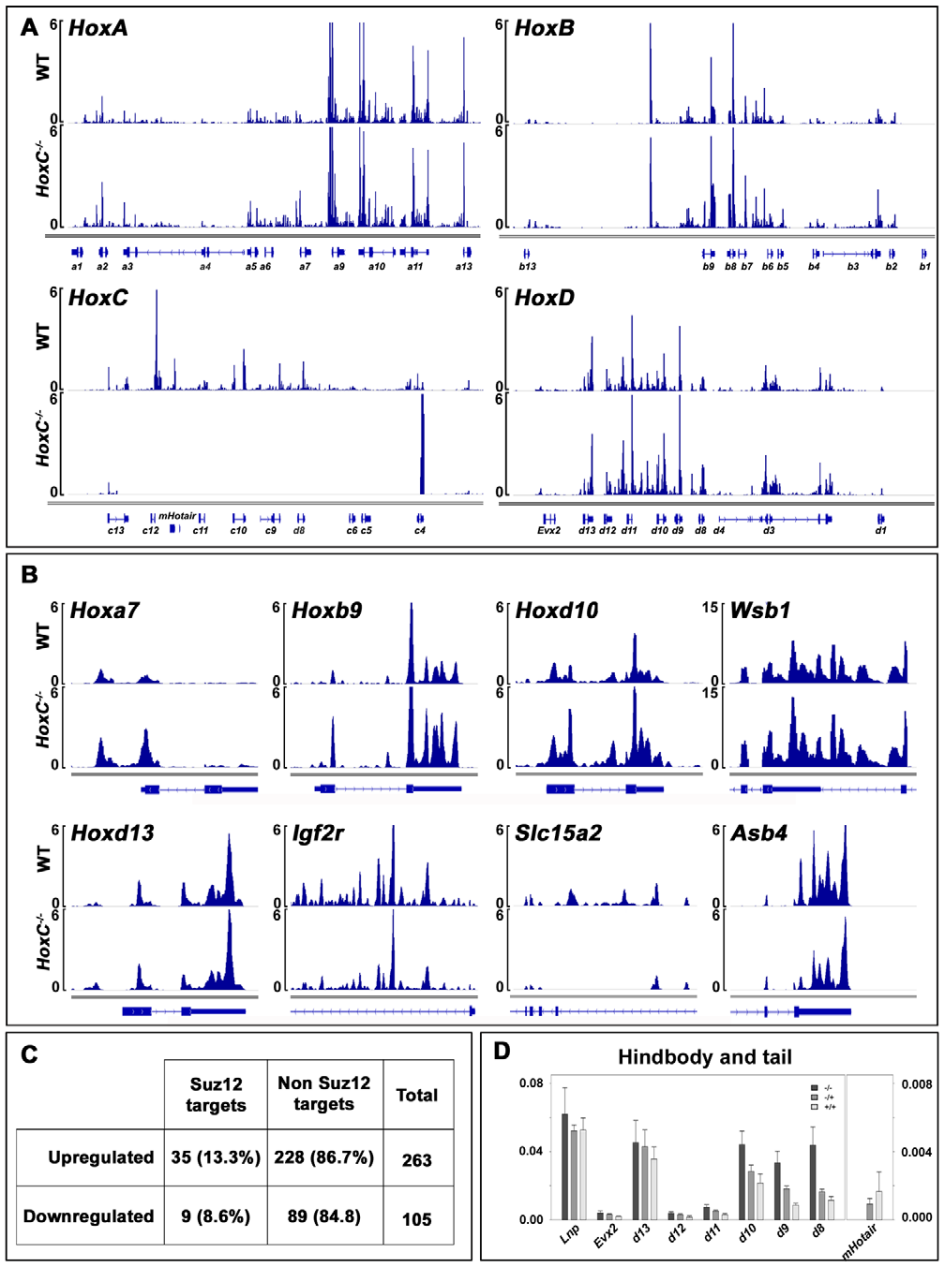
RNA–seq profiles of control and *HoxC* mutant mice. RNA was extracted from the region enriched in *mHotair* transcripts at day 13.5, i.e. the posterior part of the fetus, including the tail, hindlimbs and the outgrowing genitalia. Plotted are mean values of 25 bp windows. (A) Transcription profiles of the four different *Hox* gene clusters. The positions of the genes are indicated below. (A) Expression profiles of all four *Hox* loci, shown with the orientation with respect to the centromers. The strong peak in the deleted *HoxC* cluster is a transcript induced over the second exon of *Hoxc4* (non-deleted) after deletion of the cluster (see the text). (B) Examples of transcriptional variations induced by the deletion of the *HoxC* cluster, with some genes being slightly up-regulated (*Hoxa7*, *Hoxb9*, *Hoxd10* and *Wsb1*), some being down-regulated (*Igf2r*, *Slc15a2*, *Asb4*). *Hoxd13* is shown as an unaffected control gene (*Hoxd13*). (C) Percentage of genes either up-regulated or down-regulated in *HoxC* mutant animals, which were also reported to be the targets of SUZ12 in ES cells. The percentages are comparable, suggesting that capacity to recruit PRC2 may not be the main cause of the transcriptional variations observed in the *HoxC* mutant animals, in these tissues at this developmental time. (D) Absolute quantifications of posterior *Hoxd* gene transcripts and of *mHotair* in posterior parts of fetuses including the hindlimbs, the genital bud and the developing tail of E11.5 embryos. All values are normalized to a housekeeping gene.

We compared mutant and wild type transcript profiles genome wide and observed significant changes. These modifications, which may reflect direct or indirect targets either of *mHotair*, or of *Hoxc* gene products, were either up- or down regulated and broadly distributed over all gene ontology categories. *Hox* genes were included, along with housekeeping genes and genes from unrelated structures and functions (Figure 4A and 4B). We looked at the *HoxD* cluster and the strongest variation in steady-state level of transcripts was observed for *Hoxd8*, *Hoxd9*, *Hoxd10* and *Hoxd11*, as previously reported for HOTAIR in human cells, though the amplitudes were significantly lower (Figure 4A). While these results appeared at first to somehow correlate with the reported effect of human HOTAIR on this gene cluster, Rinn *et al.* [10] observed a substantial increase in expression of these genes by down-regulating HOTAIR by a factor of two thirds, whereas we detected a maximum of three-fold difference in the complete absence of this lincRNA.

To assess whether these differences could be partly explained by the relatively low expression of *mHotair* at this particular stage (E13.5) or a dilution effect, we isolated RNA from the same set of tissues, i.e. hindbody, hindlimbs and genital bud, from E11.5 embryos and quantified the RNAs by reverse transcription PCR. Differences in absolute expression levels of the different *Hox* genes analyzed were comparable to those obtained in our RNA-seq experiment at E13.5, suggesting that the observed effects of *mHotair* and *HoxC* deletions on gene regulation are reproducible, at least between these two developmental stages (Figure 4D).

The discrepancies between our results and those reported previously may reflect a dilution effect due to only few cells expressing *mHotair* in our samples. However, we also observed a slight up-regulation of *Hoxd1*, *Hoxd3* and *Hoxd4* and, surprisingly, our mutants exhibited no change in *Hoxd13* transcripts (Figure 4B and 4D), neither in downstream-located non coding RNAs, a region significantly up-regulated in previous work. Also, we observed a similar de-repression of *Hox* genes belonging to other clusters, with *Hoxa7* and *Hoxb9* showing comparable up-regulations (two fold, Figure 4B), unlike previously reported. Of note, a substantial increase of transcripts matching the second exon of *Hoxc4*, i.e. the most 3′ part remaining after the deletion of the *HoxC* gene cluster. This unexpected burst likely reflects the presence of ‘posterior-acting’ regulation, which are now re-routed towards this sequence, in the absence of the intervening *HoxC* cluster, as describe in similar contexts [30]. Taken together, while these observations support a general, though rather moderate, effect of removing the *HoxC* gene cluster, including *mHotair*, in the posterior part of the developing embryo, transcriptome analyses confirmed the difficulty to attribute to *mHotair* the same regulatory capacities during embryonic development, than those associated to its human counterpart in cultured fibroblasts.

Even though the structure of *mHotair* showed substantial differences with its human ortholog, we looked for additional evidence of a potential role as a molecular scaffold to bridge PcG proteins to their target sites. We assessed whether or not the group of genes that displayed a clear transcriptional de-repression in *HoxC* mutant animals was enriched in genes known to recruit PRC2 in ES cells, i.e. in conditions where *Hox* clusters are covered by H3K27me3. We applied a stringent cut-off with a significance window of 1 kb and obtained 263 genes up-regulated in the mutant sample, whereas 105 genes were down-regulated. We looked at which fraction of these genes represented known PcG targets, as defined by binding to SUZ12 [31]. Of the 263 genes defined as up-regulated in the *HoxC* null mice, only 35 (13%) had been determined as being bound by SUZ12 in ES cells (Figure 4C). Likewise, out of a total of 105 genes down-regulated, only 16 were bound by SUZ12 (15%), a figure that was down to 8.6% after *Hoxc* genes were removed from the list (since they are deleted in the mutant) (Figure 4C).

## Discussion

The importance of long non-coding RNAs (lincRNAs) for gene regulation has been recently emphasized in many different contexts. One of the paradigms of this novel class of transcripts is the human HOTAIR RNA, which is encoded from within the *HOXC* gene cluster and acts in *trans* to regulate *HOXD* target genes *via* the recruitment of PRC2 and further tri-methylation of H3K27 [10]. Interestingly, the mouse counterpart shows little sequence conservation with HOTAIR. While such lincRNAs are known to be moderately conserved in sequence between different species, sequence alignment between the mouse and human *HoxC* clusters reveals that the DNA fragments included in both HOTAIR and *mHotair* are amongst the less conserved within the *Hoxc12* to *Hoxc11* DNA interval, as if they would correspond to the less constrained sequences in terms of evolution. Yet some intron-exon borders are conserved, as well as the direction of transcription, which suggests that the mouse *HoxC* cluster does contain a genuine cognate HOTAIR RNA.

Interestingly, the first three exons of HOTAIR seem to be absent from *mHotair*, which appears to contain two exons only, a first exon related to the fourth exon of HOTAIR, followed by a larger exon 2, related to the large sixth exon of HOTAIR. Even though an increase in the number of sequence reads may reveal the presence of either additional, poorly spliced 5′ located exons or alternative start sites, *mHotair* is thus quite distinct in structure from its human cognate. Such a divergence may underlie important differences in function since the first three exons of HOTAIR (absent from *mHotair*) contain binding sites for EZH2. Likewise, the LSD1 binding sequences, localized at the 3′ extremity of human HOTAIR, is part of the least conserved DNA sequence within *mHotair* exon 2 (below 70% conservation). Altogether, based on DNA sequence analyses, it is difficult to reconcile the structure of *mHotair* with the potential function previously attributed to HOTAIR, even though binding of both EZH2 and LSD1 proteins may mostly rely on tri-dimensional structures rather than upon specific RNA sequences.

This conclusion was re-enforced by the expression analyses during mouse development, which revealed patterns different from those previously reported when a human probe was used to assess the presence of mouse transcripts [10]. As expected, *mHotair* is expressed very much like the neighboring *Hoxc11* gene, i.e. in parts of the proximal hindlimbs, in the posterior part of the body and in the emerging presumptive external genital organs. We think that this discrepancy in expression patterns can be explained by the very low sequence conservation between the human RNA antisense probe and the mouse target RNA. Coordinated expression of RNA or transgenes introduced within *Hox* gene clusters has been reported in several instances [32] and illustrates the strong global regulation that controls these groups of genes. Non-*Hox* promoters located in- or introduced into- these loci tend to adopt the shared expression specificities and thus behave like their nearest neighbors.

The genetic ablation of *mHotair*, under physiological condition, confirmed the apparent difference between the functions of this lincRNA in mice and humans. Firstly, *Hoxd* genes expression remained moderately affected in most tissues analyzed, as assessed by quantitative PCR, *in situ* hybridization and RNA-seq, in particular in those tissues of the developing body where steady-state levels of *mHotair* were the highest. Secondly, the group of genes that was either up- or down-regulated in the absence of *mHotair*, as scored by transcriptome analyses, did not particularly overlap with known PcG targets as described in ES cells, nor was it enriched in any of the GO terms. Thirdly, no significant difference was scored in the amount of H3K27me3 decorating the *HoxD* locus, neither by using embryonic tissues, nor when assessing MEFs derived from homozygous null fetuses. This latter point may reflect the fact that mouse *Hotair* lacks most of the cognate human 5′ RNA fragment, which was shown to be necessary for the binding of EZH2 [21]. Although we cannot exclude that the deletion of *mHotair* may have induced a subtle effect upon *Hox* gene expression, these genes would need to be affected much more severely for a phenotypic outcome to be observed, as animals heterozygous for a deletion of the entire *HoxD* cluster are virtually of wild type phenotype [33]. Therefore, only a robust impact of *mHotair* on *Hoxd* genes regulation would make this lincRNA a candidate regulator of these developmental genes in mice, at least at the time when critical changes in chromatin status are observed [22].

How can we explain this unexpected difference in the functional importance of cognate non-coding RNAs in two mammalian species where both the structure and function of *Hox* genes appear to be highly conserved? First, our mutant configuration not only lacks *mHotair*, but also all *Hoxc* genes as well as the potential mouse ortholog of FRIGIDAIR, another lincRNA located within the *HoxC* cluster [3] and whose deletion could counterbalance the effect of removing *mHotair*. However, our transcriptome dataset indicates that *mFrigidair*, if present in the mouse genome, is not transcribed at detectable level in our posterior body sample, unlike *mHotair*, which makes this possibility unlikely. Also, there is no evidence supporting a strong effect of HOXC proteins over *Hoxd* genes regulation. If any, this effect would need to exactly compensate for a potential effect of *mHotair* such that the situation in the mutant samples would look like wild type.

Secondly, the function of *mHotair* could be restricted to a limited number of cells within the expression domains of *Hox* genes, in which case our selection of a rather large piece of tissue would reduce the sensitivity of our functional assays *via* a dilution effect, which would not occur in cultured fibroblasts. While this is a serious possibility, it would imply that only a small subset of *Hox* positive cells would be ‘exposed’ to *mHotair*, questioning its general importance in the recruitment of PRC2 during development. Alternatively, human HOTAIR may be required for *HOXD* gene regulation at later stages and in different contexts, rather than in the early recruitment of PRC2 over the *HOXD* cluster. As for all other posterior *Hox* genes, *Hoxc11* and *Hoxc12* expression is restricted towards the posterior part of the developing body in early mouse embryos. It is nonetheless conceivable that *mHotair* be transcribed subsequently, in a tissue or organ where it may have a functional importance, such as in foreskin fibroblasts where its function was originally described. This would imply that the recruitment of PRC2 and subsequent tri-methylation of H3K27 over *Hoxd* cluster genes would be achieved by different mechanisms in different contexts or, at least, by using various components to recruit PRC2.

Another possibility is that *mHotair* and HOTAIR may have importantly diverged and no longer share any functional similarity. Non-coding RNAs are generally rather poorly conserved in sequences amongst different species and this possibility may not be overtly surprising. The fact that RNA sequences present in HOTAIR and associated with the binding of either EZH2 or LSD1 do not seem to be present in *mHotair* supports this view. However, this would be difficult to reconcile with HOTAIR being a key player in the regulation of *HOX* genes in human, since this gene family has been the paradigm of the structural and functional conservation of genetic circuitries in vertebrates, not talking about mammals.

Alternatively, *mHotair* may have a genuine function in organizing the chromatin landscape over *Hox* genes, but its deletion *in vivo* could activate redundant or compensatory pathways still allowing proper PcG-mediated silencing to occur, a mechanism absent from cultured human fibroblasts. Silencing of *Hox* genes during early development must be tightly achieved, to prevent precocious activation leading to mis-identification of structures. Yet this repression will have to be easily reversed subsequently, in the many different contexts where these genes will be activated. Whether or not this epigenetic versatility would be best implemented by redundant silencing mechanisms or by a preponderant strategy relying upon PRC2 dependent tri-methylation of H3K27 is difficult to evaluate. In both cases, *mHotair* may be recruited to the *HoxD* cluster to help this silencing to be established, in those regions where it is expressed. However, our results argue against this mechanism being a fundamental process in *Hox* gene silencing, in particular as these gene clusters are tightly covered by PcG proteins and decorated by tri-methylated H3K27 in all embryonic contexts analyzed so far where these genes must be repressed, i.e. mostly in tissues where *mHotair* transcripts were below our detection level.

## Materials and Methods

### Ethics statement

All experiments involving living animals were authorized by- and carried out following- the swiss legal framework.

### Mutant mice

Mice carrying a deletion of the *HoxC* gene cluster were published previously [26]. They were purchased from the RIKEN BioResource Center (BRC), in Japan. Heterozygous mice were crossed to obtain wild type, heterozygous and homozygous mutant embryos. Genotyping was performed on individual yolk sacs with the following primers:

WTforward: CGCTCTGGGAGTGGTCTTCAGAAG;
WTreverse: GTGCTACGATCTGTTATGTATGTG;
delCforward: GATGGAGTTTCCCCACACTGAGTG;
delCreverse: CGTGAGGAAGAGTTCTTGCAGCTC.

### Sequence comparison

Sequences alignments between the mouse and human *HoxC* loci were performed using the pairwise Lagan analysis from the Vista website [23].

### *In situ* hybridization

Mid-day of vaginal plug was considered as E0.5. Embryos were dissected in PBS and fixed overnight at 4° in 4% PFA. Whole mount *in situ* hybridization was performed according to standard protocols. The decreasing signal intensity observed for the oldest processed embryos is partially due to the somewhat lower permeability of the probe, along with tissue differentiation. Mutant, heterozygous and wild type animals were processed simultaneously to ensure identical conditions. The *Hoxd10* probe was as previously described [34]. The murine *Hotair* and *Hoxc11* probes were PCR-subcloned into pGEM-T Easy vector (Promega), sequence verified, linearized and *in vitro* transcribed with either SalI-T7 (antisense) or NcoI-SP6 (sense), using the DIG RNA Labeling Mix (Roche).

*mHotair* forward: GAGCCAGAGCTGAAGGTATG
*mHotair* reverse: AAGACACGCACGGAGAAAGG
*Hoxc11* forward: CCCCGCACCCGCAAGAAGC
*Hoxc11* reverse: GTCCAGTTTTCCACCCGCGG

### Chromatin immunoprecipitation

Chromatin immunoprecipitation followed by quantitative reverse transcription was performed as previously described [35]. Briefly, cells or tissues were fixed for 15 minutes in 1% formaldehyde, washed three times in cold PBS and stored at −80° before being processed using polyclonal anti-H3K27me3 antibody (Millipore, 17-622).

### Cell culture

Mouse embryonic fibroblasts were derived from heterozygous crosses of E13.5 embryos using standard protocols. Cells were cultured in MEF culture conditions in DMEM supplemented with 10% FBS. Isolated lines were first genotyped using tissues from the embryos and subsequently confirmed with DNA extraction procedures. Passage No 4 MEFs were used for further experiments.

### Expression analysis

The posterior parts of embryos including the hindlimbs, the genital bud and the developing tail at day 11.5 and the forebody, hindbody, forelimbs and hindlimbs at day 13.5, were dissected and stored in RNAlater (Qiagen) until genotyped. Cells or tissues were first disrupted and homogenized using a Polytron (kinematic) before RNA was extracted using the RNeasy Microkit (Qiagen, 74034), followed by qRT-PCR with SYBR Green. Mean values derive from two (MEFs) or four (tissues) biological replicates, processed in triplicates and normalized to a housekeeping gene (*Rps9*).

### RNA–seq and downstream analysis

The most posterior parts of fetuses at day E13.5 were dissected, including the hindlimbs, the genital bud and the developing tail, and total RNA was extracted as for expression analysis. Wild type and mutant samples were deep sequenced using the Illumina Genome Analyzer. Reads were mapped onto the mouse mm9 genome using Tophat and visualized with the integrative genome viewer (mean value of 25 bp windows). Mis-regulated genes were identified using a 200 bp binning approach across the genome. Significance was measure by the presence of probes showing a difference between wt and mutant profiles greater than 6 over at least 5 probes (1 kb).
